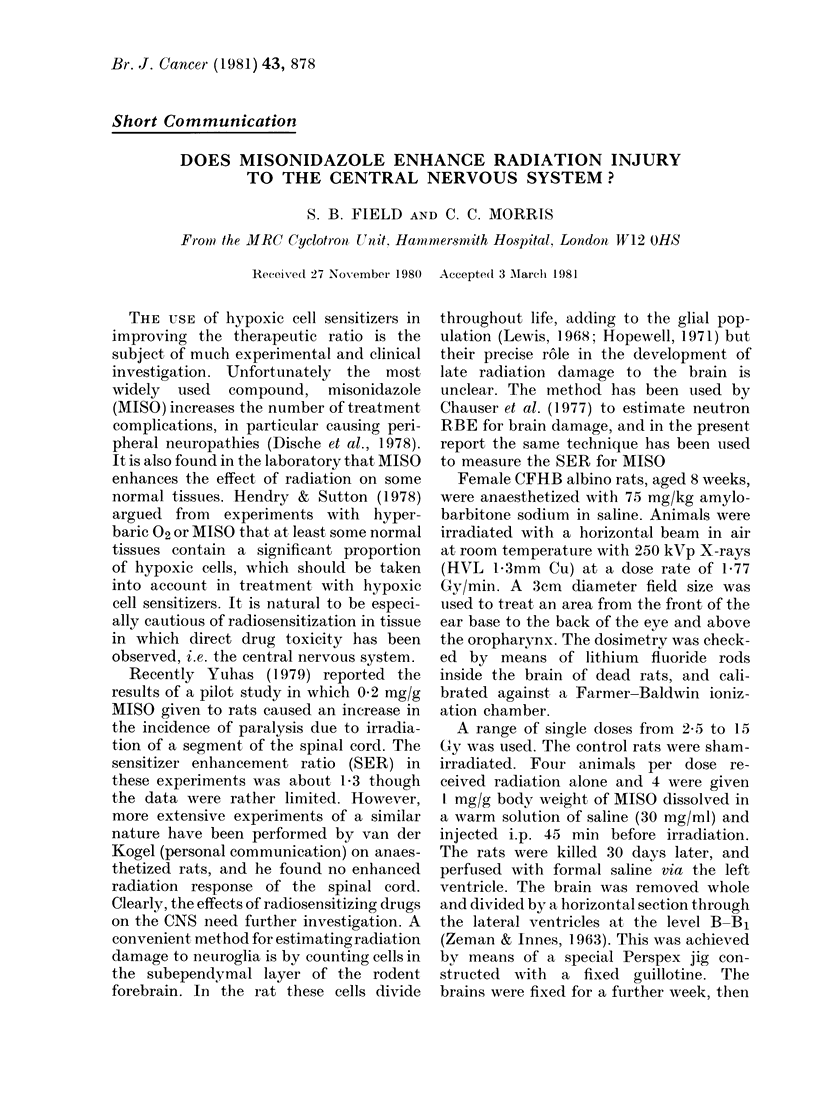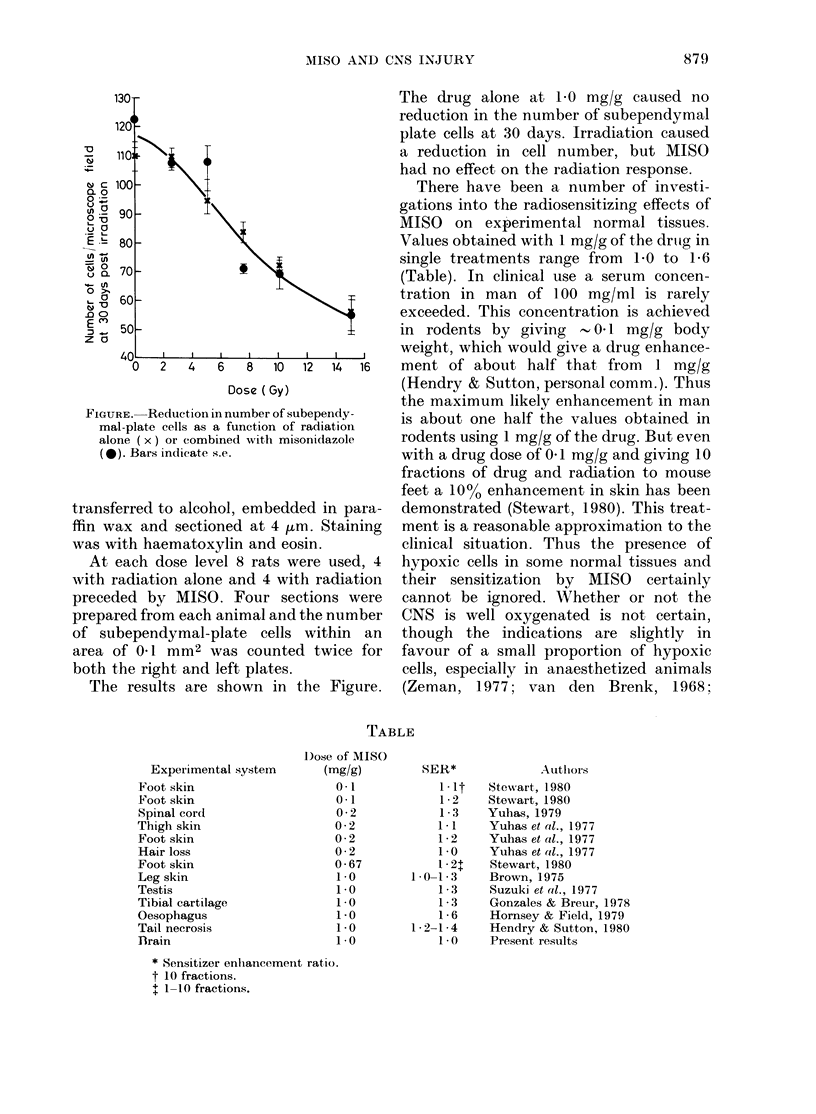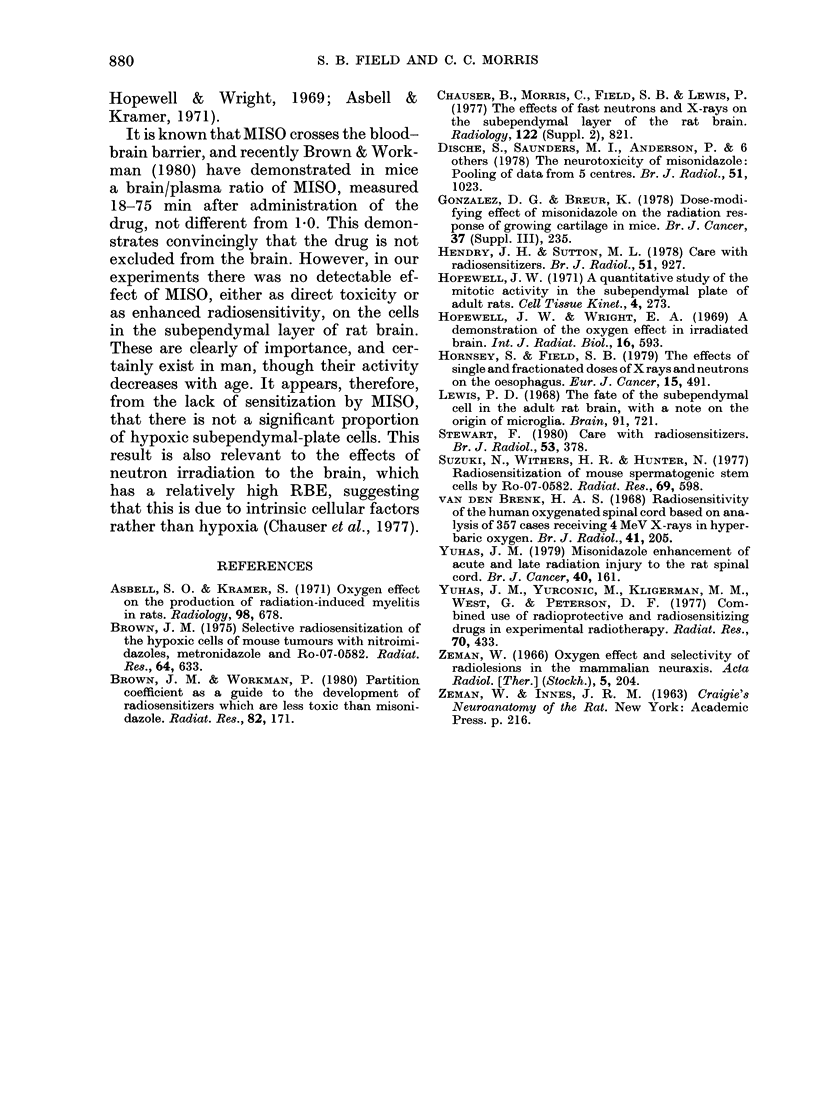# Does misonidazole enhance radiation injury to the central nervous system?

**DOI:** 10.1038/bjc.1981.128

**Published:** 1981-06

**Authors:** S. B. Field, C. C. Morris


*Br. J. Cancer* (1981) **43**, 878

***Short Communication***

### DOES MISONIDAZOLE ENHANCE RADIATION INJURY TO THE CENTRAL NERVOUS SYSTEM?

S. B. FIELD AND C. C. MORRIS

*From the MRC Cyclotron Unit, Hammersmith Hospital, London W12 0HS*

Received 27 November 1980 Accepted 3 March 1981

THE USE of hypoxic cell sensitizers in improving the therapeutic ratio is the subject of much experimental and clinical investigation. Unfortunately the most widely used compound, misonidazole (MISO) increases the number of treatment complications, in particular causing peripheral neuropathies (Dische *et al.*, 1978). It is also found in the laboratory that MISO enhances the effect of radiation on some normal tissues. Hendry & Sutton (1978) argued from experiments with hyperbaric $O_2$ or MISO that at least some normal tissues contain a significant proportion of hypoxic cells, which should be taken into account in treatment with hypoxic cell sensitizers. It is natural to be especially cautious of radiosensitization in tissue in which direct drug toxicity has been observed, *i.e.* the central nervous system.

Recently Yuhas (1979) reported the results of a pilot study in which 0·2 mg/g MISO given to rats caused an increase in the incidence of paralysis due to irradiation of a segment of the spinal cord. The sensitizer enhancement ratio (SER) in these experiments was about 1·3 though the data were rather limited. However, more extensive experiments of a similar nature have been performed by van der Kogel (personal communication) on anaesthetized rats, and he found no enhanced radiation response of the spinal cord. Clearly, the effects of radiosensitizing drugs on the CNS need further investigation. A convenient method for estimating radiation damage to neuroglia is by counting cells in the subependymal layer of the rodent forebrain. In the rat these cells divide throughout life, adding to the glial population (Lewis, 1968; Hopewell, 1971) but their precise rôle in the development of late radiation damage to the brain is unclear. The method has been used by Chauser *et al.* (1977) to estimate neutron RBE for brain damage, and in the present report the same technique has been used to measure the SER for MISO

Female CFHB albino rats, aged 8 weeks, were anaesthetized with 75 mg/kg amylobarbitone sodium in saline. Animals were irradiated with a horizontal beam in air at room temperature with 250 kVp X-rays (HVL 1·3mm Cu) at a dose rate of 1·77 Gy/min. A 3cm diameter field size was used to treat an area from the front of the ear base to the back of the eye and above the oropharynx. The dosimetry was checked by means of lithium fluoride rods inside the brain of dead rats, and calibrated against a Farmer–Baldwin ionization chamber.

A range of single doses from 2·5 to 15 Gy was used. The control rats were sham-irradiated. Four animals per dose received radiation alone and 4 were given 1 mg/g body weight of MISO dissolved in a warm solution of saline (30 mg/ml) and injected i.p. 45 min before irradiation. The rats were killed 30 days later, and perfused with formal saline *via* the left ventricle. The brain was removed whole and divided by a horizontal section through the lateral ventricles at the level $B\text{–}B_1$ (Zeman & Innes, 1963). This was achieved by means of a special Perspex jig constructed with a fixed guillotine. The brains were fixed for a further week, then

FIGURE.—Reduction in number of subependymal-plate cells as a function of radiation alone (×) or combined with misonidazole (●). Bars indicate s.e.

transferred to alcohol, embedded in paraffin wax and sectioned at 4 $\mu$m. Staining was with haematoxylin and eosin.

At each dose level 8 rats were used, 4 with radiation alone and 4 with radiation preceded by MISO. Four sections were prepared from each animal and the number of subependymal-plate cells within an area of 0·1 $mm^2$ was counted twice for both the right and left plates.

The results are shown in the Figure. The drug alone at 1·0 mg/g caused no reduction in the number of subependymal plate cells at 30 days. Irradiation caused a reduction in cell number, but MISO had no effect on the radiation response.

There have been a number of investigations into the radiosensitizing effects of MISO on experimental normal tissues. Values obtained with 1 mg/g of the drug in single treatments range from 1·0 to 1·6 (Table). In clinical use a serum concentration in man of 100 mg/ml is rarely exceeded. This concentration is achieved in rodents by giving ~0·1 mg/g body weight, which would give a drug enhancement of about half that from 1 mg/g (Hendry & Sutton, personal comm.). Thus the maximum likely enhancement in man is about one half the values obtained in rodents using 1 mg/g of the drug. But even with a drug dose of 0·1 mg/g and giving 10 fractions of drug and radiation to mouse feet a 10% enhancement in skin has been demonstrated (Stewart, 1980). This treatment is a reasonable approximation to the clinical situation. Thus the presence of hypoxic cells in some normal tissues and their sensitization by MISO certainly cannot be ignored. Whether or not the CNS is well oxygenated is not certain, though the indications are slightly in favour of a small proportion of hypoxic cells, especially in anaesthetized animals (Zeman, 1977; van den Brenk, 1968;

TABLE

| Experimental system | Dose of MISO (mg/g) | SER* | Authors |
|---|---|---|---|
| Foot skin | 0·1 | 1·1† | Stewart, 1980 |
| Foot skin | 0·1 | 1·2 | Stewart, 1980 |
| Spinal cord | 0·2 | 1·3 | Yuhas, 1979 |
| Thigh skin | 0·2 | 1·1 | Yuhas *et al.*, 1977 |
| Foot skin | 0·2 | 1·2 | Yuhas *et al.*, 1977 |
| Hair loss | 0·2 | 1·0 | Yuhas *et al.*, 1977 |
| Foot skin | 0·67 | 1·2‡ | Stewart, 1980 |
| Leg skin | 1·0 | 1·0–1·3 | Brown, 1975 |
| Testis | 1·0 | 1·3 | Suzuki *et al.*, 1977 |
| Tibial cartilage | 1·0 | 1·3 | Gonzales & Breur, 1978 |
| Oesophagus | 1·0 | 1·6 | Hornsey & Field, 1979 |
| Tail necrosis | 1·0 | 1·2–1·4 | Hendry & Sutton, 1980 |
| Brain | 1·0 | 1·0 | Present results |

* Sensitizer enhancement ratio.

† 10 fractions.

‡ 1–10 fractions.

Hopewell & Wright, 1969; Asbell & Kramer, 1971).

It is known that MISO crosses the blood–brain barrier, and recently Brown & Workman (1980) have demonstrated in mice a brain/plasma ratio of MISO, measured 18–75 min after administration of the drug, not different from 1·0. This demonstrates convincingly that the drug is not excluded from the brain. However, in our experiments there was no detectable effect of MISO, either as direct toxicity or as enhanced radiosensitivity, on the cells in the subependymal layer of rat brain. These are clearly of importance, and certainly exist in man, though their activity decreases with age. It appears, therefore, from the lack of sensitization by MISO, that there is not a significant proportion of hypoxic subependymal-plate cells. This result is also relevant to the effects of neutron irradiation to the brain, which has a relatively high RBE, suggesting that this is due to intrinsic cellular factors rather than hypoxia (Chauser *et al.*, 1977).

### REFERENCES

Asbell, S. O. & Kramer, S. (1971) Oxygen effect on the production of radiation-induced myelitis in rats. *Radiology*, **98**, 678.

Brown, J. M. (1975) Selective radiosensitization of the hypoxic cells of mouse tumours with nitroimidazoles, metronidazole and Ro-07-0582. *Radiat. Res.*, **64**, 633.

Brown, J. M. & Workman, P. (1980) Partition coefficient as a guide to the development of radiosensitizers which are less toxic than misonidazole. *Radiat. Res.*, **82**, 171.

Chauser, B., Morris, C., Field, S. B. & Lewis, P. (1977) The effects of fast neutrons and X-rays on the subependymal layer of the rat brain. *Radiology*, **122** (Suppl. 2), 821.

Dische, S., Saunders, M. I., Anderson, P. & 6 others (1978) The neurotoxicity of misonidazole: Pooling of data from 5 centres. *Br. J. Radiol.*, **51**, 1023.

Gonzalez, D. G. & Breur, K. (1978) Dose-modifying effect of misonidazole on the radiation response of growing cartilage in mice. *Br. J. Cancer*, **37** (Suppl. III), 235.

Hendry, J. H. & Sutton, M. L. (1978) Care with radiosensitizers. *Br. J. Radiol.*, **51**, 927.

Hopewell, J. W. (1971) A quantitative study of the mitotic activity in the subependymal plate of adult rats. *Cell Tissue Kinet.*, **4**, 273.

Hopewell, J. W. & Wright, E. A. (1969) A demonstration of the oxygen effect in irradiated brain. *Int. J. Radiat. Biol.*, **16**, 593.

Hornsey, S. & Field, S. B. (1979) The effects of single and fractionated doses of X rays and neutrons on the oesophagus. *Eur. J. Cancer*, **15**, 491.

Lewis, P. D. (1968) The fate of the subependymal cell in the adult rat brain, with a note on the origin of microglia. *Brain*, 91, 721.

Stewart, F. (1980) Care with radiosensitizers. *Br. J. Radiol.*, **53**, 378.

Suzuki, N., Withers, H. R. & Hunter, N. (1977) Radiosensitization of mouse spermatogenic stem cells by Ro-07-0582. *Radiat. Res.*, **69**, 598.

van den Brenk, H. A. S. (1968) Radiosensitivity of the human oxygenated spinal cord based on analysis of 357 cases receiving 4 MeV X-rays in hyperbaric oxygen. *Br. J. Radiol.*, **41**, 205.

Yuhas, J. M. (1979) Misonidazole enhancement of acute and late radiation injury to the rat spinal cord. *Br. J. Cancer*, **40**, 161.

Yuhas, J. M., Yurconic, M., Kligerman, M. M., West, G. & Peterson, D. F. (1977) Combined use of radioprotective and radiosensitizing drugs in experimental radiotherapy. *Radiat. Res.*, **70**, 433.

Zeman, W. (1966) Oxygen effect and selectivity of radiolesions in the mammalian neuraxis. *Acta Radiol.* [*Ther.*] (*Stockh.*), **5**, 204.

Zeman, W. & Innes, J. R. M. (1963) *Craigie's Neuroanatomy of the Rat*. New York: Academic Press. p. 216.